# Adaptation and psychometric testing of the end-of-life professional caregiver survey in Jamaica

**DOI:** 10.1186/s12913-023-09497-2

**Published:** 2023-05-16

**Authors:** Rebecca L. Edwards, Marie Bakitas, Peng Li, Dingle Spence, Eulalia Kahwa, Mark Stoltenberg, Nataliya V. Ivankova, Kaesha Thomas, Kammar Segree, Syed Matthew Kodilinye, Adelais Markaki

**Affiliations:** 1 Department of Acute, Chronic, and Continuing Care, School of Nursing, University of Alabama at Birmingham, 1720 2nd Avenue South, Birmingham, AL 35294-1210 US; 2 School of Nursing, University of Alabama at Birmingham, Birmingham, AL US; 3 Hope Institute Hospital, 7 Golding Ave, Kingston 7, Mona, Jamaica; 4 Department of Medicine, University of the West Indies, Mona Campus, Mona, Jamaica; 5 Jamaica Cancer Care and Research Institute, University of the West Indies, Mona Campus, Mona, Jamaica; 6 School of Health and Behavioral Sciences, University of the West Indies, Five Islands Campus, Antigua and Barbuda, Jamaica; 7 Global Palliative Care Program, Division of Palliative Care and Geriatrics, Massachusetts General Hospital, Boston, Massachusetts US; 8 Harvard Medical School, Boston, Massachusetts US; 9 Department of Health Services Administration, School of Health Professions, University of Alabama at Birmingham, Birmingham, AL US; 10 PAHO/WHOCC for International Nursing, Family, Community and Health Systems Department, School of Nursing, University of Alabama at Birmingham, Birmingham, AL US

**Keywords:** Palliative care, Hospice, End-of-life, Interprofessional, Education, Psychometrics, Validity, Low- and middle-income countries

## Abstract

**Background:**

Using a validated instrument to measure palliative care (PC) educational needs of health professionals is an important step in understanding how best to educate a well-versed PC workforce within a national health system. The End-of-life Professional Caregiver Survey (EPCS) was developed to measure U.S. interprofessional PC educational needs and has been validated for use in Brazil and China. As part of a larger research project, this study aimed to culturally adapt and psychometrically test the EPCS among physicians, nurses, and social workers practicing in Jamaica.

**Methods:**

Face validation involved expert review of the EPCS with recommendations for linguistic item modifications. Content validation was carried out by six Jamaica-based experts who completed a formal content validity index (CVI) for each EPCS item to ascertain relevancy. Health professionals practicing in Jamaica (n = 180) were recruited using convenience and snowball sampling to complete the updated 25-item EPCS (EPCS-J). Internal consistency reliability was assessed using Cronbach’s $\alpha$ coefficient and McDonald’s $\phi$. Construct validity was examined through confirmatory factor analysis (CFA) and exploratory factor analysis (EFA).

**Results:**

Content validation led to elimination of three EPCS items based on a CVI < 0.78. Cronbach’s $\alpha$ ranged from 0.83 to 0.91 and McDonald’s $\phi$ ranged from 0.73 to 0.85 across EPCS-J subscales indicating good internal consistency reliability. The corrected item-total correlation for each EPCS-J item was > 0.30 suggesting good reliability. The CFA demonstrated a three-factor model with acceptable fit indices (RMSEA = 0.08, CFI = 0.88, SRMR = 0.06). The EFA determined a three-factor model had the best model fit, with four items moved into the *effective patient care* subscale from the other two EPCS-J subscales based on factor loading.

**Conclusions:**

The psychometric properties of the EPCS-J resulted in acceptable levels of reliability and validity indicating that this instrument is suitable for use in measuring interprofessional PC educational needs in Jamaica.

## Background

The World Health Organization (WHO) defines palliative care (PC) as *“a crucial part of integrated, people-centered health services. Relieving serious health-related suffering, be it physical, psychological, social, or spiritual, is a global ethical responsibility. Thus, whether the cause of suffering is cardiovascular disease, cancer, major organ failure, drug-resistant tuberculosis, severe burns, end-stage chronic illness, acute trauma, extreme birth prematurity or extreme frailty of old age, PC may be needed and has to be available at all levels of care”* [1, para 1]. Using an interprofessional team approach, PC aims to relieve the physical symptoms, psychosocial distress, and spiritual pain from the point of diagnosis for any patient with a serious illness [2]. The United Nations (UN) and the WHO include PC as a fundamental human right and a critical component of the Sustainable Development Goals (SDGs) [3] and Universal Health Coverage (UHC) [4]. These global goals call for equitable access to PC services for all patients and their families facing serious illness. Despite these advanced, only 14% of those in need across the globe have access to PC [1]. Hence, to make progress towards the goals of UHC and achieving the SDGs, it is imperative to strengthen PC services.

Like most low- and middle-income countries (LMICs), access to Jamaica’s PC services remains extremely limited. According to the Global Atlas of Palliative Care [5], Jamaica is Category 3a, meaning there is only *isolated PC provision*. As highlighted by the WHO public health model for the integration of PC, advances in policy, education, medication access, and service implementation are all necessary to expand access to comprehensive PC. Of these four pillars, a 2010 Jamaican needs assessment specifically identified lack of education as a key area of need for the country [6]. The 67th World Health Assembly recommended PC education and training for all hospital- and community-based healthcare professionals to *strengthen PC as a component of comprehensive care throughout the life course* [7]. These recommendations called for *basic* PC education as a routine element of all undergraduate medical and nursing education, *intermediate* education for all healthcare professionals who routinely care for patients with serious illnesses across settings, and *specialist-*level education for interprofessional PC team members who manage the most complex patients [8].

A key step in improving PC education programs is to first identify the educational needs of healthcare professionals in the country. Prior to this study, existing surveys to measure discipline-specific PC educational needs had not been validated for use in Jamaica [9–13]. The End-of-Life Professional Caregiver Survey (EPCS) was designed to measure PC educational needs across healthcare disciplines. The EPCS was initially developed and validated in the U.S. [14], and was later adapted for use in Brazil [15] and China [16]. The original EPCS has 28 items across three subscales, *Patient and Family-Centered Communication, Cultural and Ethical Values*, and *Effective Care Delivery*, rated on a five-point Likert scale. A low EPCS score indicates higher PC educational needs [14].

Given the ECPS’ utility across disciplines, cultural adaptability, and psychometric rigor, this tool was selected to ascertain interprofessional PC educational needs in Jamaica. This study aimed to culturally adapt and psychometrically test the EPCS for use in Jamaica by determining face, content, and construct validity, as well as reliability. It also serves as part of a larger research project that investigates ways to educate a well-versed PC workforce in Jamaica.

## Methods

### Study design

EPCS validation comprised a three-phase process: *Phase I* determined face validity, *Phase II* determined content validity, and *Phase III* implemented the adapted survey (EPCS-J) to determine psychometric properties in a Jamaican context, including internal consistency, reliability, and construct validity. In *Phase I*, we recruited five Jamaican study team members who evaluated the EPCS for face validity and integrated linguistic survey modifications. In *Phase II*, we recruited a panel of six Jamaican PC experts who used a formal content validity process [17] to obtain an item-level content validity index (I-CVI) for EPCS items. In *Phase III*, the modified EPCS-J was distributed to physicians, nurses, and social workers who practice in Jamaica and survey results were statistically analyzed to determine psychometric properties. Findings from all phases are reported based on the Strengthening the Reporting of Observational Studies in Epidemiology (STROBE) checklist for cross-sectional studies [18].

### Settings and participants

Jamaica’s national health system has four Regional Health Authorities [19, 20]. Kingston, the capital city, is in the Southeast Regional Health Authority (SERHA). For *Phase I*, five SERHA-based PC experts, who were also study team members, reviewed the EPCS survey for face validity. For *Phase II*, study team members identified six Jamaica-based interprofessional PC experts to participate in a content validity process of the EPCS. Inclusion of six experts allowed for use of Lynn’s criteria for retaining survey items with an I-CVI of no lower than 0.78 [20].

For *Phase III*, we implemented the survey across Jamaica’s four Regional Health Authorities. A convenience sample of participants was identified by a multi-modal recruitment strategy [21]. Study team members recruited in-person participants across SERHA healthcare settings using a Qualtrics [22] generated QR code shared to mobile devices, and by posting and distributing recruitment flyers. Additionally, multiple healthcare professional organizations serving all of Jamaica’s health regions were asked to electronically distribute surveys via their email listservs, using a Qualtrics-generated hyperlink. Inclusion criteria were: (1) healthcare professionals practicing in Jamaica including general and specialty physicians; assistant, general, and specialist nurses; and social workers who cared for patients with late-stage serious illnesses, (2) at least 19 years of age, and (3) English-speaking.

### Instrument

The original EPCS contained 28-items across three subscales: *Patient and Family-Centered Communication, Cultural and Ethical Values*, and *Effective Care Delivery*. The first subscale contained 12 items, and the other two subscales contained 8 items each. Responses were measured on a five-point Likert scale (0 = *not at all*; 1 = *a little bit*; 2 = *somewhat*; 3 = *quite a bit*; and 4 = *very much*). Scores ranged from 0 to 112 with lower scores indicating higher PC educational needs [14]. Nine *participant characteristic* items (gender, ethnic background, age, healthcare discipline, educational level, years in professional practice, exposure to PC training, healthcare setting, and practice location), and 12 *PC educational preferences* items were also included on the survey.

#### Phase I: EPCS face Validity

Two Jamaica-based co-investigators, a PC specialist physician (DS), and a professor of nursing (EK) reviewed the survey and made recommended linguistic item modifications to match the cultural context. Once the recommended changes were made, the U.S.-based investigators met with four members of the Jamaica-based study team and discussed additional survey item adaption to best meet a Jamaican context.

#### Phase II: EPSC Content Validation

We created a *Content Validity* survey, modeled after Lynn’s technique [20]. The survey included the 28 EPCS survey items on a four-point Likert scale to determine item relevance (1 = not relevant, 2 = somewhat relevant, 3 = quite relevant, 4 = highly relevant). A four-point scale was used to avoid neutral or ambivalent midpoint scores [17, 20]. Our Jamaica-based study team members identified six PC content experts to complete the survey; three physicians, two nurses, and one social worker who represented the target population and had expertise across disciplines. Each of the six respondents rated the items and provided narrative comments pertaining to items requiring changes. After content validation, three items were eliminated leaving a final survey (EPCS-J) comprised of 25 items.

#### Phase III: Survey and Psychometric Testing

The 25-item EPCS-J was entered to the Qualtrics software platform [22] for ease of distribution and statistical analyses. Our goal was to recruit at least 10 participants per retained EPCS-J item ($$n=250)$$ [23, 24]. Three Jamaica-based research assistants led recruitment efforts. On-the-ground recruitment efforts were focused on healthcare institutions across the SERHA region. Participants were asked to complete surveys on their mobile devices using a Qualtrics-generated QR code. Healthcare professionals unable to complete the surveys, due to time or other constraints, were offered a study flyer with a QR code to access the survey at their convenience. One Jamaica-based study team member (KT) led efforts to distribute surveys via organizational email listservs (Table 1).

**Table 1:** Healthcare professional organizations targeted for survey distribution by discipline

Discipline	Organization
Medicine	Medical Association of Jamaica
	Jamaica Medical Doctors Association
	Association of General Practitioners of Jamaica
	Medical Council of Jamaica
	Caribbean College of Family Physicians
	Kingston and St. Andrew Primary Care Doctors Association
Nursing	Nurses Association of Jamaica Nursing Council of Jamaica
	Jamaica Association of Nurse Practitioners
Social Work	Jamaica Association of Social Workers

**Fig. 1:**
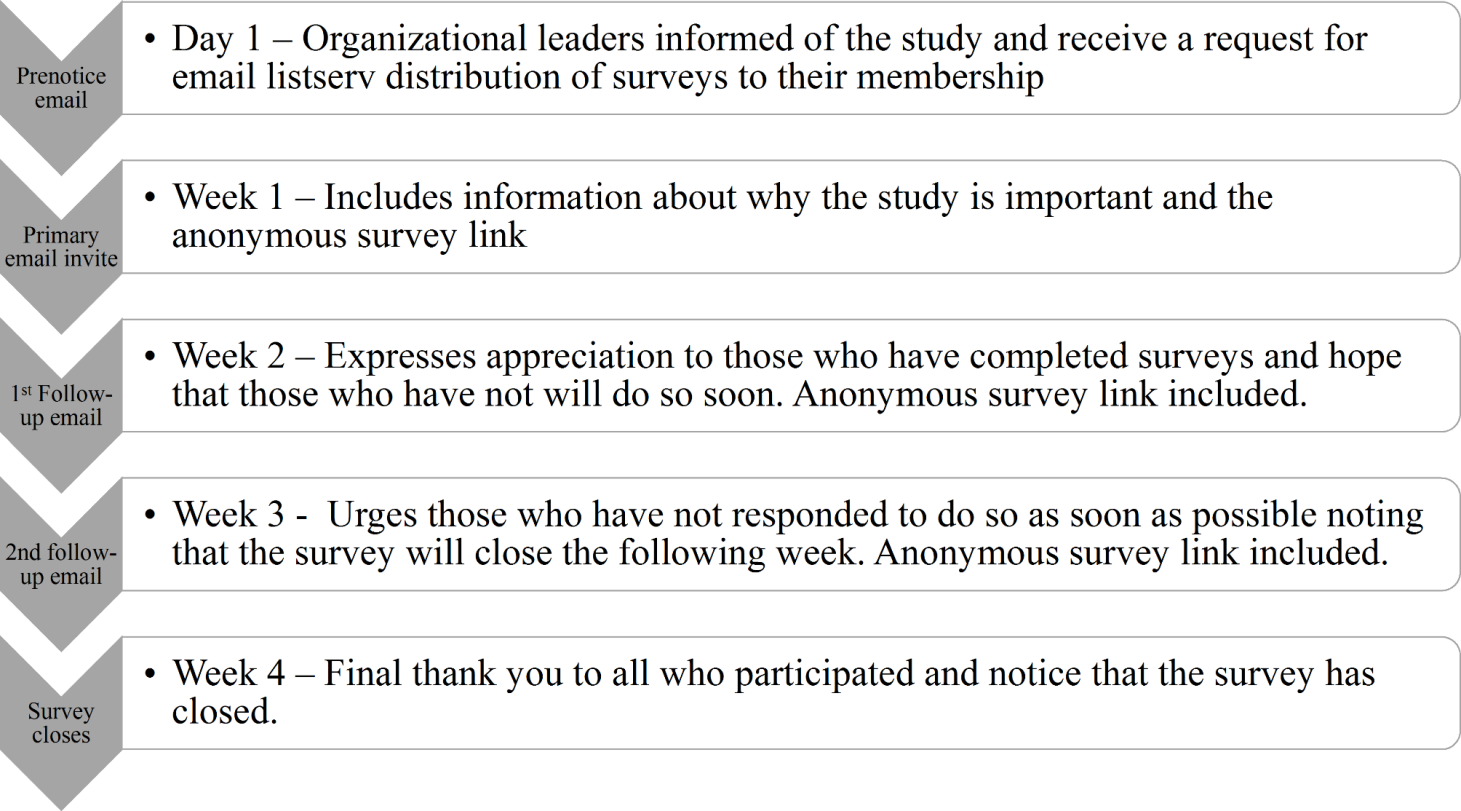
Modified Dillman method of survey distribution to maximize responses

Each organization’s administrator was contacted via email using a cover letter outlining the study objectives and rationale. Copies of the Jamaica-based ethics committee approvals were attached to each email. A modified version of Dillman’s method to maximize survey response rates was incorporated for email recruitment (Fig. 1) [25].

Once agreement to distribute surveys was obtained, subsequent emails to organizational administrators included study information and a Qualtrics-generated hyperlink which allowed survey access. Administrators were asked to distribute this information to their email listservs using the modified Dillman method timeline [25]. During collection, the primary investigator monitored survey responses and electronically distributed a $25US gift card incentives as part of the agreement for survey completion. The survey was initially distributed, using these methods, between April and June 2022.

Due to low survey uptake, we kept the survey open for another ten weeks. During these weeks, those who received the survey link through healthcare professional organization email listserv distribution had additional time to complete the survey. Jamaica-based research assistants continued to recruit participants on-the-ground. We incorporated snowball sampling via email messages to those who completed the survey requesting that they distribute the survey to other eligible participants in their networks. Also, study investigators contacted health professional leaders at Jamaica’s Ministry of Health and Wellness, the University Hospital of the West Indies, and the University of the West Indies requesting that they distribute the survey to eligible health professionals. We compared participants’ demographics and contact information to ensure survey responses were unique. The survey was closed August 14, 2022.

### Ethical considerations

Ethical approval of the study protocol was obtained from the Mona Campus Research Ethics Committee of the University of the West Indies (CREC-MN.200 20/21), Jamaica’s Ministry of Health and Wellness (2020/55), and the University of Alabama at Birmingham’s Institutional Review Board (IRB-300007515-001). The Qualtrics-based survey was prefaced with a greeting comprising study-related information and a consent form. Participants gained survey access after reviewing the consent information and selecting the *‘I agree’* option. Participants were informed that the study was voluntary, and they were free to withdraw whenever they wished. Surveys were housed on the primary investigator’s password-protected computer and Qualtrics-based security mechanisms were optimized to protect participant anonymity and confidentiality. Participants had the opportunity to share their names and email addresses for purposes of obtaining an electronic gift card incentive for survey completion and were informed that this information would be destroyed once gift cards were distributed.

### Data analysis

In *Phase II*, we used Microsoft® Excel for Mac Version 16.61.1 to calculate I-CVIs based on ratings offered by the six experts. Item scores 3 or 4 were considered *‘relevant’* and 1 or 2 *‘irrelevant’* thus dichotomizing the ordinal scale. An I-CVI was computed by dividing the number of relevant responses by the total number of experts. An I-CVI less than 0.78 indicated irrelevance [26].

In *Phase III*, we used R software to clean, code and analyze the survey data. Internal consistency reliability was evaluated using Cronbach’s $\alpha$and McDonald’s $\omega$ for the three subscales. The subscales represented the three survey dimensions: *patient and family-centered communication* (items P1 - P3, P5 - P12); *cultural and ethical values* (items C1 - C8); and *effective care delivery* (items E2 – E5, E7 - E8). Construct validity of the survey was ascertained using a confirmatory factor analysis (CFA) and an exploratory factor analysis (EFA). The model fit for the CFA was determined using the standardized root mean squared residual (SRMR), the root mean-square error of approximation (RMSEA), and the comparative fit index (CFI). Model fit with SRMR < 0.08, CFI > 9.0, and RMSEA < 0.06 was used to determine if the three survey dimensions were retained in the Jamaican context.

## Results

### Phases I and II

In *Phase I*, the face validation process led to EPCS modifications based on recommendations by study team members. For instance, we added ‘mixed Caribbean’ and ‘Black Caribbean’ selections to the Ethnic Background item, changed ‘code status’ to ‘resuscitation status’ in one of the *patient- and family-centered communication* EPCS items, and modified ‘continuing credit hours’ to ‘continuing education hours’ in the *educational preferences* items.

In *Phase II*, the six content experts completed the survey and offered narrative comments for item adjustments. We calculated I-CVIs for each of the 28 EPCS items. This resulted in elimination of three of the 28 EPCS items, one from the *patient- and family-centered communication* subscale, and two from the *effective care delivery* subscale. Remaining items were linguistically modified based on expert suggestions. For instance, Item P1 was modified from *I am comfortable helping patients and families to understand a poor prognosis* to *I am comfortable helping patients and families to understand the meaning of a poor prognosis*, and item C3 was modified from *I am comfortable caring for dying patients* to *I am comfortable (mentally, spiritually) caring for dying patients*. The original EPCS items, I-CVIs, expert comments, and modified EPCS-J items can be found in Table 2.

**Table 2:** Content Validation of EPCS Items

Survey Items	CVI	Expert comments	Item revision or elimination
Patient- and Family-Centered Communication subscale			
P1. I am comfortable helping patients and families to understand a poor prognosis.	1.00	I am comfortable explaining to patients and their families, what it means to have a poor prognosis? Or I am comfortable helping patients, and their families understand the meaning of their prognosis?	I am comfortable helping patients and families to understand the meaning of a poor prognosis.
P2. I am able to assist patients with serious illness and families set goals for care.	1.00	I am able to assist patients and their families set patient-centered goals of end-of-life care or set goals of care along the disease trajectory?	I am able to assist patients with serious illness and families set goals for care along the disease trajectory including end-of-life care.
P3. I am comfortable talking to patients and families about personal choice and self-determination.	1.00	None	
P4. I am comfortable starting and participating in discussions about resuscitation status.	0.67	I am comfortable discussing life-sustaining treatments with patients and their families? -I am comfortable starting and participating in discussions about resuscitation measures.	Eliminated
P5. I can assist family members and others through the grieving process.	1.00	None	
P6. I am able to document patient needs and suggested patient care interventions.	1.00	None	
P7. I am comfortable talking with other health professionals about the care of dying patients.	1.00	The meaning of this question isn’t clear to me… it’s too open to interpretation.	
P8. I am comfortable helping to resolve family conflicts about end-of-life care.	0.83	None	
P9. I can recognize impending death (physiologic changes).	1.00	None	
P10. I know how to use non-drug therapies to manage patient symptoms.	1.00	I think two separate questions should be asked about knowledge and comfort. I know how to use non- drug therapies to prevent and manage patient symptoms or …to use non- drug therapies for (optimum) patient symptom control?	
P11. I am able to address patients’ and family members’ fears of getting addicted to pain medications.	1.00	None	
P12. I encourage patients and families to complete advance care planning (such as a living will).	0.83	None	
Cultural and Ethical Considerations subscale			
C1. I am comfortable dealing with ethical issues related to end-of-life/hospice/palliative care.	1.00	None	
C2. I am able to deal with my feelings related to working with dying patients.	1.00	This question is too open to interpretation. Dealing with feelings may translate to an expert avoidant or substance misuse or abuse for an emotionally unintelligent health care provider. I’m trying to think of a suggestion…	I am able to effectively deal with my feelings (maintain self-care) related to working with dying patients.
C3. I am comfortable caring for dying patients.	1.00	I am unsure of what “comfortable caring” as a competence means… Do you mean comfort as in psychological competence (as in the previous question) or knowledge or combined skillset?	I am comfortable (mentally, spiritually) caring for dying patients.
C4. I am comfortable assessing how spiritual issues can impact the care of patients with serious illness and their families.	1.00	None	
C5. I am comfortable assessing how spiritual issues can impact the care of patients with serious illness and their families.	1.00	This is a repeat question I believe.	I am comfortable dealing with patients’ and families’ religious and spiritual perspectives
C6. I am comfortable providing emotional support to grieving families.	1.00	None	
C7. I am comfortable providing emotional support to grieving staff members.	1.00	None	
C8. I am knowledgeable about cultural factors influencing end-of-life care.	1.00	None	
Effective Patient Care subscale			
E1. I can recognize when patients should be navigated to a hospice center for terminal care.	0.67	These facilities are close to non-existent in Jamaica	Eliminated
E2. I am familiar with palliative care principles that guide health professional palliative care education.	1.00	None	
E3. I am effective at helping patients and families navigate the healthcare system.	1.00	None	
E4. I am familiar with the services provided by the hospice facilities in Jamaica.	1.00	None	
E5. I am effective at helping to maintain continuity across care settings.	1.00	None	
E6. I am confident addressing requests for assisted suicide.	0.67	I think the word " confident” confers some judgement and may prevent full honesty in responding. How about “comfortable” or I know how to address requests for… Does not align with Jamaican culture/legal	Eliminated
E7. I have personal resources to help meet my needs when working with dying patients and families.	1.00	I am not clear on the meaning of this question	
E8. My workplace provides resources to support staff who care for dying patients.	1.00	None	

### Phase III

#### Sample characteristics

**Fig. 2:**
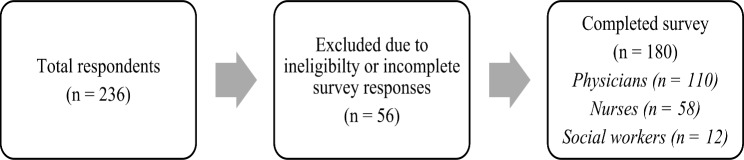
Sampling flowchart

In total, 236 healthcare professionals responded to the survey. After excluding those with incomplete responses $\left(n=56\right)$, 180 surveys were analyzed representing 110 physicians (61.1%), 58 nurses (32.2%), and 12 social workers (6.7%) **(**Fig. 2**)**.

The response rate could not be calculated because the number of healthcare professionals who received emailed surveys was unknown. Although we sought to identify the numbers of professional organization members to whom the survey was sent, these were not available. The sociodemographic characteristics of the sample are presented in Table 3.

**Table 3:** Sociodemographic of the study population$(n=180)$

Variable	N (%)
**Gender**	
Male Female Prefer not to answer	25 (13.9) 154 (85.6) 1 (0.5)
**Ethnic background**	
Black Caribbean Mixed Caribbean Other	130 (72.2) 24 (13.3) 26 (14.5)
**Age (years)**	
19 to 24 25 to 34 35 to 44 45 to 54 55 to 64 65 or older	5 (2.8) 106 (58.9) 37 (20.6) 19 (10.6) 8 (4.4) 5 (2.8)
**Healthcare profession**	
Generalist physician Specialist physician Generalist nurse Specialist nurse Nurse practitioner Nurse midwife Social worker	75 (41.7) 35 (19.4) 38 (21.1) 10 (5.6) 5 (2.8) 5 (2.8) 12 (6.7)
**Level of Education**	
Certificate Diploma Bachelor’s degree Master’s degree PhD Post-doctoral training Other (e.g., Doctor of Medicine)	8 (4.4) 8 (4.4) 114 (63.3) 29 16.1) 3 (1.7) 13 (7.2) 5 (2.8)
**Time in profession**	
< 1 year 1 to 5 years 6 to 10 years 11 to 15 years 16 to 20 years 21 years or more	3 (1.7) 87 (48.3) 33 (18.3) 15 (8.3) 17 (9.4) 25 (13.9)
**Palliative care training**	
Yes No	46 (25.6) 134 (74.4)
**Setting**	
Public hospital Private hospital Public health center Private clinic Patients’ homes Long term care - rehabilitation Other	105 (58.3) 14 (7.8) 19 (10.6) 21 (11.7) 11 (6.1) 2 (1.1) 8 (4.4)
**Location**	
Urban Rural or remote Both urban and rural/remote	119 (66.1) 22 (12.2) 39 (21.7)

Respondents were predominantly female (86%), of mixed Caribbean ethnicity (72%), between 25 and 34 years of age (59%), had bachelor’s degrees (63%), with 1 to 5 years of experience (48%), had never received formal PC or end-of-life care training (74%), and primarily practiced in public hospitals (66%) in urban settings (58%).

#### Reliability

The corrected item-total correlation coefficients, Cronbach’s $\alpha$, and McDonald’s $\omega$ for each subscale are presented in Table 4. For the 25-item survey, corrected item-total correlations ranged from 0.39 to 0.82, indicating high correlation of items to each subscale and high reliability [27]. Cronbach’s $\alpha$ was 0.91 for the *patient- and family-centered communication* subscale, 0.91 for the *cultural and ethical values* subscales, and 0.83 for the *effective care delivery* subscale, indicating good internal consistency and reliability across all three subscales. This was substantiated with McDonald’s $\omega$, which was 0.85 for the *patient- and family-centered communication* subscale, 0.80 for the *cultural and ethical values* subscale, and 0.73 for the *effective care delivery* subscale. The Cronbach’s $\alpha$ value did not change much following exclusion of any item across all three subscales. The mean scores and standard deviations for the three subscales and for each survey item are presented in Table 4.

**Table 4:** Summary of reliability and mean scores and standard deviations for EPCS items$(n=25)$

Items (No. of items)	Corrected Item-Total Correlation	Cronbach’s Alpha if Item Deleted	Cronbach’s alpha	McDonald’s Omega	Mean (SD)
**(P) Patient- and family-centered communication (11)**			**0.91**	**0.85**	2.40 (0.81)
P1 I am comfortable helping patients and families to understand the meaning of a poor prognosis	0.60	0.91			2.73 (1.03)
P2 I am able to assist patients with serious illness and families set goals for care along the disease trajectory including end-of-life care	0.82	0.89			2.13 (1.20)
P3 I am comfortable talking to patients and families about personal choice and self-determination	0.70	0.90			2.50 (1.15)
P5 I can assist family members and others through the grieving process	0.72	0.90			2.37 (1.09)
P6 I am able to document patient needs and suggested patient care interventions	0.71	0.90			2.79 (1.03)
P7 I am comfortable talking with other health professionals about the care of dying patients	0.70	0.90			2.92 (1.04)
P8 I am comfortable helping to resolve family conflicts about end-of-life care	0.76	0.90			1.76 (1.21)
P9 I can recognize impending death (physiologic changes)	0.39	0.92			2.96 (0.96)
P10 I know how to use non-drug therapies to manage patient symptoms	0.64	0.91			1.98 (1.11)
P11 I am able to address patients’ and family members’ fears of getting addicted to pain medications	0.72	0.90			2.13 (1.11)
P12 I encourage patients and families to complete advance care planning (such as a living will)	0.62	0.91			1.78 (1.32)
**(C) Cultural and Ethical Values (8)**			**0.91**	**0.80**	0.86 (0.53)
C1 I am comfortable dealing with ethical issues related to end-of-life/hospice/palliative care	0.70	0.90			2.00 (1.21)
C2 I am able to effectively deal with my feelings (maintain self-care) related to working with dying patients	0.67	0.90			2.68 (1.11)
C3 I am comfortable (mentally, spiritually) caring for dying patients	0.70	0.90			2.58 (1.15)
C4 I am comfortable assessing how spiritual issues can impact the care of patients with serious illness and their families	0.71	0.89			2.56 (1.08)
C5 I am comfortable dealing with patients’ and families’ religious and cultural perspectives	0.68	0.90			2.61 (1.04)
C6 I am comfortable providing emotional support to grieving families	0.76	0.89			2.48 (1.10)
C7 I am comfortable providing emotional support to grieving staff members	0.73	0.89			2.38 (1.07)
C8 I am knowledgeable about cultural factors influencing end-of-life care	0.66	0.90			2.24 (1.07)
**(E) Effective Care Delivery (6)**			**0.83**	**0.73**	0.84 (0.46)
E2 I am familiar with palliative care principles that guide health professional palliative care education	0.66	0.79			1.68 (1.10)
E3 I am effective at helping patients and families navigate the healthcare system	0.60	0.81			2.36 (1.01)
E4 I am familiar with the services provided by the hospice facilities in Jamaica	0.64	0.80			1.62 (1.23)
E5 I am effective at helping to maintain continuity across care settings	0.71	0.79			2.07 (1.10)
E7 I have personal resources to help meet my needs when working with dying patients and families	0.55	0.82			1.41 (1.19)
E8 My workplace provides resources to support staff who care for dying patients	0.48	0.83			0.94 (1.19)

#### Construct validity

The CFA goodness of fit indices revealed $RMSEA of 0.08$, $CFI of 0.88$, and $SRMR of 0.06,$ suggesting acceptable fit (Table 5**).**

**Table 5:** Confirmatory factor analysis fits for the three EPCS-J subscales

Index	Index Criteria	Fit Index in Jamaican Sample
RMSEA (CI)	$<0.06$	0.08 (acceptable fit)
CFI	$> 0.90$	0.88 (acceptable fit)
SRMR	$> 0.08$	0.06 (good fit)

RMSEA Root mean square error of approximation, CI confidence interval, CFI Comparative fit index, SRMR Standardized root mean square residual.

To further explore the structural validity of the EPCS-J in a Jamaican sample, an EFA was performed. Based on eigenvalues > 1; a scree plot; and a parallel analysis; two-, three-, and four-factor models were considered possible. Here, we only report the three-factor model based on the best model fit (RMSR = 0.04). The results from the EFA suggested that items P9 (*I can recognize impending death - physiologic changes*), P10 (*I know how to use non-drug therapies to manage patient symptoms*), and P11 (*I am able to address patients’ and family members’ fears of getting addicted to pain medications*) had higher loadings within the *effective patient care* subscale rather than in the *patient- and family-centered communication subscale*, where they originated. Furthermore, C1 *(I am comfortable dealing with ethical issues related to end-of-life/hospice/palliative care*) had higher loadings within the *effective patient care* subscale, rather than in the *cultural and ethical considerations* subscale, where it originated. After regrouping items based on the three-factor model, the internal consistency reliability based on Cronbach’s $\alpha$ increased, ranging from 0.89 to 0.90, and was similar to the original model based on McDonald’s $\omega$, ranging from 0.77 to 0.82. Table 6 presents factor loadings for all EPCS-J items.

**Table 6:** Loading value from the three-factor EFA for EPCS-J items and mean of each item

Factor	Original EPCS-J item number	Factor loading			Mean (SD)
		Factor 1	Factor 2	Factor 3	
(P) Patient- and family-centered communication (11)					
	P3	0.28	0.27	**0.70**	2.50 (1.15)
	P2	0.47	0.28	**0.66**	2.13 (1.20)
	P1	0.14	0.23	**0.64**	2.73 (1.03)
	P8	0.51	0.26	**0.58**	1.76 (1.21)
	P5	0.35	0.46	**0.54**	2.37 (1.09)
	P6	0.40	0.34	**0.52**	2.79 (1.03)
	P7	0.32	0.39	**0.51**	2.92 (1.04)
	P12	0.34	0.23	**0.46**	1.78 (1.32)
	P11	**0.47**	0.29	0.44	2.13 (1.11)
	P10	**0.53**	0.33	0.31	1.98 (1.11)
	P9	**0.31**	0.15	0.22	2.96 (0.96)
**(C) Cultural and Ethical Values (8)**					
	C3	0.28	**0.72**	0.19	2.58 (1.15)
	C2	0.25	**0.67**	0.20	2.68 (1.11)
	C5	0.21	**0.63**	0.31	2.61 (1.04)
	C4	0.26	**0.66**	0.28	2.56 (1.08)
	C6	0.33	**0.59**	0.40	2.48 (1.10)
	C7	0.38	**0.53**	0.36	2.38 (1.07)
	C8	0.45	**0.47**	0.25	2.24 (1.07)
	C1	**0.60**	0.38	0.46	2.00 (1.21)
**(E) Effective Care Delivery (6)**					
	E2	**0.66**	0.27	0.35	1.68 (1.10)
	E5	**0.64**	0.24	0.31	2.36 (1.01)
	E4	**0.62**	0.21	0.22	1.62 (1.23)
	E3	**0.55**	0.22	0.35	2.36 (1.01)
	E7	**0.51**	0.35	0.06	1.41 (1.19)
	E8	**0.42**	0.29	0.23	0.94 (1.19)

## Discussion

As part of a larger effort, this study modified and determined the utility of the EPCS in Jamaica. The 25-item EPCS-J was found to be psychometrically sound with acceptable reliability and validity when measuring the PC educational needs of physicians, nurses, and social workers in Jamaica.

Content validation resulted in removal of three items from the original EPCS based on CVI < 0.78. Item P4 had to do with resuscitation status. In Jamaica, *do not resuscitate* (DNR) orders exist but are not widely recognized among healthcare professionals, so removal of this item was culturally appropriate. Item E1 had to do with navigating patients to hospice for terminal care. In Jamaica, there is no formal hospice insurance program equivalent to what is offered in the US. Additionally, there are only two recognized inpatient hospice facilities, one in the Kingston area and one in Montego Bay. Removing this item was appropriate based on the concept of hospice care not aligning with the clinical realities of practice in Jamaica. Item E6 referred to addressing requests for assisted suicide, a practice that remains illegal throughout Jamaica. Thus, removing this item was culturally appropriate. Although these items were removed, investigating these topics (DNR, hospice navigation, and assisted suicide) warrant future investigation, possibly through qualitative study.

The three-factor EPCS-J showed satisfactory internal consistency, with a total Cronbach’s $\alpha$ of 0.91 and McDonald’s $\omega$ of 0.85. These values align with those found in prior validation studies in the U.S. [14], Brazil [15], and China [16]. Therefore, this EPCS-J version can be used to measure the PC educational needs of interprofessionals in Jamaica with good internal consistency reliability.

The CFA results indicated satisfactory construct validity. The fit indices across the model were acceptable. The RMSE was 0.08, CFI 0.88, and SRMR 0.06 indicating satisfactory goodness-of-fit when compared to the original construct. These indices were like those determined in the Garcia et al. [15] efforts to adapt and validate the EPCS for use in Brazil where they determined an RMSE of 0.8, CFI of 0.96, and SRMR of 0.06. The original U.S.-based EPCS has a rigorous structure, and the Jamaican EPCS-J maintained its validity.

The EFA confirmed that all three EPCS factors should be retained. This aligns with former validation studies of the EPCS [14, 16]. However, Garcia et al. determined only two factors (renamed *care effectiveness* and *mourning and ethical and cultural values*) should be retained for EPCS use in Brazil [15]. In our analyses, factor loading across all 25 EPCS-J items was acceptable. These results indicated that this version of the EPCS-J is suitable for use in Jamaica. However, three items originally in the *patient- and family-centered communication* subscale best fit in the *effective patient care* subscale. These items included P8 *I am comfortable helping to resolve family conflicts about end-of-life care*, P9 *I can recognize impending death (physiologic changes)*, and P10 *I know how to use non-drug therapies to manage patient symptoms*. And one item originally in the *cultural and ethical considerations* subscale best fit in the *effective patient care* subscale. This item was *C1 I am comfortable dealing with ethical issues related to end-of-life/hospice/palliative care.* Thus, these four items should be relocated to the *effective patient care* subscale when the EPCS-J is used for future studies.

### Implications

Although this study identified a valid and reliable measure, more work is needed to understand best ways to educate a competent interprofessional PC workforce in Jamaica. Specifically, quantitative analyses of EPCS-J findings should include measures of association to determine sociodemographic factors predictive of high and low EPCS-J scores. This inquiry should be supplemented with qualitative inquiry into interprofessional PC educational experiences and educational needs. Use of mixed methods research methodology could lead to discovery of facilitators and barriers to PC education among and across physician, nurse, and social worker groups in Jamaica as well as development of country-specific PC education recommendations. Future educational offerings should include resource-stratified PC education and service delivery guidelines based on resources available in Jamaica [28]. The development and dissemination of tailored PC educational offerings is a critical step in improving PC in Jamaica.

### Strengths and limitations

The major contribution of this study is the adaptation of the first measure with adequate internal consistency reliability and construct validity of the EPCS-J in a Jamaican context. Since it is unknown if respondents were representative across all settings in Jamaica, generalizability and interpretation of results should be made with caution. Among healthcare professional disciplines represented in our sample, physicians ($$n=110)$$ were the majority, followed by nurses ($$n=58)$$, then social workers ($$n=12)$$. Therefore, interpretation of nurses’ and social workers’ PC educational needs may be less robust. Although the sample size ($$n=180)$$ did not reach the recommended sample size of 250 based on 10 participants per survey item, it was adequate for survey validation. Additionally, survey respondents were predominantly young and in their early career, which may have led to an overestimation of PC education competency, as younger learners are more likely to have been exposed to PC content in their prior training than older health professionals. Last, a response rate could not be calculated based on lack of access to listserv survey distribution data. Despite these limitations, the EPCS-J addresses an important gap in health services research in Jamaica – the ability to measure interprofessional PC educational needs for building PC workforce capacity in Jamaica.

## Conclusions

Globally, universal access to integrated PC across the serious illness trajectory is recognized as a human right and an integral part of UHC [7, 29–32]. Jamaica represents a LMIC, where 78% of the unmet PC needs exist [1]. In LMICs, there is a general lack of awareness among policy makers, healthcare professionals, and the public about what PC is and its benefits for patient and health system outcomes [33]. A key barrier to overcoming the unmet need for PC is the lack of high-quality training at the basic, intermediate, and advance levels for healthcare interprofessionals. The 67th World Health Assembly Resolution 67.19 specifically urges countries *to include PC as an integral component of the ongoing education and training offered to care providers, in accordance with their roles and responsibilities* [7].

Given the sparse PC service delivery in Jamaica, it is imperative that healthcare professionals have at least basic competence in PC, and that PC capacity-building efforts aim to empower teams capable of delivering PC across healthcare settings while sustaining PC education and skill-building [6]. To achieve these goals, research must focus on the best strategies to educate a competent interprofessional PC workforce.

Having a reliable and valid instrument to measure interprofessional PC educational needs in Jamaica is an important step in capacity building. This study tested EPCS face and content validity, as well as internal consistency reliability and construct validity, in a Jamaican context. Findings revealed that the EPCS-J is a reliable and valid instrument to identify the PC interprofessional educational needs in Jamaica. The EPCS-J is the first validated instrument, empirically tested to measure this construct in Jamaica. This survey can be used in future PC educational settings and research. Our hope is that EPCS-J findings will steer future PC educational interventions across Jamaica that will contribute to training a well-versed PC workforce.

## Data Availability

The datasets used and analyzed during this study are available from the corresponding author upon request.

## Abbreviations

PC: Palliative care
EPCS: End-of-Life Professional Caregiver Survey
EPCS-J: End-of-Life Professional Caregiver Survey-Jamaica
CVI: Content validity index
CFA: Confirmatory factor analysis
EFA: Exploratory factor analysis
RMSEA: Root mean-square error of approximation
CFI: Comparative fit index
SRMR: Standardized root mean squared residual
WHO: World Health Organization
SDG: Sustainable Development Goals
UHC: Universal health coverage
LMIC: Low- and middle-income country
I-CVI: Item-level content validity index
STROBE: Strengthening the Reporting of Observational Studies in Epidemiology
SERHA: Southeast Regional Health Authority
